# Augmented reality–supported self-care training and clinical outcomes in patients with major depressive disorder: a retrospective propensity score–matched cohort study

**DOI:** 10.3389/fpsyt.2026.1948675

**Published:** 2026-09-14

**Authors:** Wen Zhang, Lingling Yang

**Affiliations:** 1 Integrated Chinese and Western Depression Center, Hunan Provincial Second People’s Hospital (Hunan Provincial Brain Hospital), Changsha, China; 2 Addiction Medicine Center, Hunan Provincial Second People’s Hospital (Hunan Provincial Brain Hospital), Changsha, China

**Keywords:** augmented reality, digital health, major depressive disorder, medication adherence, psychiatric nursing, self-care agency

## Abstract

**Background:**

Augmented reality (AR) has emerged as a promising educational tool in mental health care; however, evidence regarding its integration into routine psychiatric nursing practice remains limited. This study examined whether participation in an AR-supported self-care training program was associated with improved clinical and functional outcomes among patients with Major Depressive Disorder (MDD) receiving standard psychiatric nursing care.

**Methods:**

A retrospective propensity score–matched cohort study was conducted using electronic medical record data from 342 inpatients and outpatients treated at a tertiary psychiatric hospital in Changsha, Hunan, China between January 2023 and June 2025. All patients received routine psychiatric nursing services. A subgroup additionally participated in an AR-supported self-care education program focused on illness management, daily self-care, emotion regulation, and social functioning. Propensity score methods were used to balance baseline demographic and clinical characteristics between exposure groups. Primary outcomes included changes in depressive symptom severity measured by the Hamilton Depression Rating Scale (HAM-D-17) and self-care agency measured by the Exercise of Self-Care Agency (ESCA) Scale. Secondary outcomes included medication adherence and healthcare utilization indicators.

**Results:**

Within the propensity score–matched cohort, participation in the AR-supported program was associated with significantly greater improvement in self-care agency and modestly greater reduction in depressive symptoms compared with standard care alone (both p <.001). Medication adherence was also higher among AR participants. Within the AR group, the number of completed sessions was positively correlated with improvement in self-care agency (r = 0.29, p <.001), whereas the association with symptom reduction was weaker. Multivariable analyses identified AR participation as an independent predictor of both functional and clinical improvement.

**Conclusion:**

AR-supported self-care education may represent a useful adjunct to psychiatric nursing practice by enhancing patient engagement, self-management capacity, and treatment adherence among individuals with MDD. Prospective studies are needed to confirm these findings and evaluate long-term outcomes.

## 1 Introduction

Major Depressive Disorder (MDD) is a common psychiatric disorder associated with substantial disability, impaired quality of life, and disruption of social and occupational functioning. Beyond affective symptoms, many individuals with MDD experience difficulties in attention, memory, executive functioning, and other cognitive domains that can interfere with everyday activities and recovery (1). These difficulties are clinically relevant because successful management of depression extends beyond reducing depressive symptoms and often requires patients to maintain treatment-related behaviors and daily routines outside formal clinical encounters (2).

Self-management and self-care are therefore important components of sustained recovery from mood disorders (3). Patients may need to monitor symptoms, follow medication regimens, maintain daily routines, use coping strategies, and recognize early signs of deterioration (4, 5). Evidence from mood-disorder research suggests that self-help and self-management approaches may contribute to relapse prevention and longer-term recovery (4, 5). Within psychiatric nursing practice, education and support related to medication management, symptom monitoring, daily living activities, coping, and continuity of care are consequently important elements of recovery-oriented care (6). However, the ability of patients with MDD to acquire and consistently apply self-care skills may be affected by reduced motivation, impaired concentration, and cognitive difficulties (5).

Educational approaches that rely primarily on verbal explanation or written information may not always translate knowledge into observable behaviors, particularly when learning requires patients to apply information to everyday situations (7). These challenges have increased interest in educational approaches that are more interactive, contextual, and experience-based. Augmented reality (AR) combines digital information with the physical environment and can present visual, interactive, and task-oriented information in real time (8, 9). Unlike conventional psychoeducation, AR-supported learning can potentially allow patients to observe demonstrations, work through realistic scenarios, rehearse practical behaviors, and revisit information within a structured learning environment. Such characteristics may be particularly relevant for self-care education, in which the clinical goal is not simply to acquire information but to develop the capacity to apply that information in daily life (8, 9).

Research on AR and related immersive technologies in mental health has expanded considerably, but the existing literature remains heterogeneous in terms of target populations, intervention purposes, delivery approaches, and outcome measures (9–11). A systematic review of AR interventions in mental health identified applications across neurodevelopmental disorders, anxiety and phobia, psychoeducation and well-being, and procedural or pain-related care, while also highlighting heterogeneity and generally small sample sizes (10). Evidence from MDD populations has also begun to emerge with extended-reality approaches. For example, feasibility work has examined virtual reality–based behavioral activation in adults with MDD, and a subsequent randomized controlled trial evaluated extended-reality-enhanced behavioral activation in this population (12, 13). These studies suggest that immersive technologies may be useful for supporting behavioral engagement and symptom-related interventions in MDD, but they have primarily focused on behavioral activation and symptom-oriented treatment rather than structured self-care education delivered as part of routine psychiatric nursing care.

Several important questions therefore remain unresolved. First, relatively little is known about the use of AR specifically to support self-care education among adults with MDD. Much of the existing literature has focused on feasibility, acceptability, engagement, symptom-specific interventions, or educational and psychosocial outcomes rather than on patients’ ability to initiate and maintain self-care behaviors (10, 11, 14, 15). Second, evidence from routine psychiatric nursing practice is limited. Interventions evaluated in controlled or highly selected settings may not fully reflect the variability encountered in clinical practice, where patients differ in illness severity, treatment setting, educational background, digital familiarity, and engagement with care (15). Third, there is limited evidence concerning functional outcomes that are directly related to the goals of self-care education, particularly self-care agency, as well as related behaviors such as medication adherence. These gaps are important because the potential clinical value of an educational technology depends not only on whether patients can use it, but also on whether it can support meaningful self-management in everyday care. Importantly, the available literature does not appear to have specifically examined a structured AR-supported self-care education program integrated into routine psychiatric nursing care for adults with MDD, particularly with self-care agency as a functional outcome.

The choice of outcomes was guided by the expected clinical pathway of self-care education. By providing visual demonstrations, contextualized scenarios, and opportunities to rehearse practical behaviors, AR-supported education may facilitate the acquisition and application of self-care skills. Improvements in self-care agency were therefore considered a particularly relevant functional outcome, whereas depressive symptom severity provided an indicator of clinical status. Medication adherence was examined as a related treatment behavior that may be influenced by patients’ ability to understand, organize, and apply self-management strategies. Length of stay was included as an exploratory healthcare-utilization outcome to examine whether the intervention was associated with broader differences in clinical care.

Against this background, the present study examined the association between participation in a structured AR-supported self-care education program and clinical and functional outcomes among adults with MDD receiving routine psychiatric nursing care in inpatient and outpatient settings. The program was implemented as an adjunct to standard nursing education and incorporated interactive, context-based learning activities targeting illness management, daily self-care, emotion regulation, and social functioning. Using a retrospective cohort design with propensity score matching, the study sought to reduce measured baseline differences between patients who completed the AR-supported program and those who received standard psychiatric nursing education alone. The study focused on depressive symptom severity, self-care agency, medication adherence, and selected healthcare utilization outcomes to examine the potential relevance of AR-supported self-care education across clinical and functional domains under real-world conditions. Accordingly, the present study addresses this gap by evaluating a real-world, nurse-delivered AR-supported self-care education program in adults with MDD across both inpatient and outpatient settings.

## 2 Methods

### 2.1 Study design and participants

This retrospective cohort study was conducted at Hunan Provincial Second People’s Hospital (Brain Hospital of Hunan Province), a tertiary psychiatric referral center in Changsha, Hunan, China. Clinical data were extracted from the hospital’s Electronic Medical Record (EMR) system, including both Psychiatry Inpatient Wards and the Mood Disorders Outpatient Clinic, between January 1, 2023 and June 30, 2025. This period corresponded to the stable implementation phase of the hospital’s digital nursing program, which had been fully operational since November 2022.

The study aimed to examine associations between participation in an augmented reality (AR)-supported self-care education program and clinical as well as functional outcomes among patients with Major Depressive Disorder (MDD) receiving routine psychiatric nursing care. Both inpatient and outpatient populations were included to reflect real-world service delivery and to evaluate the feasibility of AR-supported education across different levels of psychiatric care. The study population consisted of adult patients diagnosed with Major Depressive Disorder according to ICD-10 diagnostic criteria as documented within the EMR system (16).

Patients were identified through systematic review of electronic clinical records and were eligible regardless of treatment setting.

Inclusion criteria were: (a) age between 18 and 60 years; (b) a baseline Hamilton Depression Rating Scale (HAM-D-17) score of 17 or greater, indicating at least moderate depressive symptom severity (Rabinowitz et al., 2022); and (c) availability of paired clinical assessments obtained at admission and discharge for inpatients or at baseline and follow-up visits for outpatients. To preserve ecological validity and reflect routine clinical practice, no minimum treatment duration was imposed. Instead, duration of care was recorded for each participant and included as a covariate in adjusted analyses.

Patients were excluded if they had any of the following: a documented psychotic disorder (e.g., schizophrenia spectrum disorders), significant neurological disease (e.g., epilepsy, neurodegenerative disorders, or traumatic brain injury), active substance dependence within the previous six months, or receipt of electroconvulsive therapy (ECT) during the observation period. These criteria were selected to minimize potential influences of severe neurocognitive impairment or intensive treatment interventions on educational engagement and outcome assessment. Patients with incomplete baseline or endpoint assessments for the primary outcome measures were excluded from the analytic cohort. Records with substantial missingness in key study variables were also excluded according to the prespecified eligibility criteria.

In addition to demographic and clinical characteristics, information regarding treatment-related variables was extracted whenever available, including psychotropic medication class, documented participation in psychotherapy, duration of illness, and duration of care. Because engagement with digital interventions may vary according to educational background and familiarity with technology, education level was included as a prespecified covariate in all adjusted analyses.

Given the retrospective and non-randomized nature of the study, findings were interpreted as associations rather than evidence of causal effects. Potential confounding factors were addressed through multivariable adjustment and additional sensitivity analyses described below.

Ethical approval for this study was obtained from the Medical Research Ethics Committee of Hunan Provincial Second People’s Hospital (Approval No: 20251105-32). The requirement for informed consent was waived because the study involved retrospective analysis of fully anonymized clinical records and posed minimal risk to participants.

### 2.2 Intervention exposure and group allocation

During the study period, the hospital’s psychiatric nursing department implemented a structured augmented reality (AR)-supported self-care education program as part of its digital nursing initiative. The program was designed to complement standard psychiatric nursing education and to support the development of practical self-management skills among patients with Major Depressive Disorder (MDD).

The intervention was delivered through the PsyCare-AR platform, a tablet-based application integrating psychoeducational content with interactive augmented reality experiences. This platform was institutionally developed specifically for the program and was not a commercially available intervention. Also, platform underwent preliminary usability and feasibility testing with psychiatric nursing staff before full clinical implementation. No formal patient-level usability or content-validation study was conducted before implementation; the preliminary feasibility assessment was limited to psychiatric nursing staff. In inpatient units, the program was accessed using hospital-issued tablet devices. Outpatients completed sessions using dedicated AR workstations located within the Mood Disorders Outpatient Clinic. The educational curriculum was developed collaboratively by psychiatric nurses, psychiatrists, and clinical psychologists and was aligned with routine nursing care objectives.

Four core modules were included: (1) Understanding depression and treatment; (2) Daily self-care and behavioral activation; (3) Emotion regulation and coping strategies; and (4) Social functioning and interpersonal communication. Each module incorporated interactive AR elements that overlaid digital information onto real-world environments. Educational strategies included visual demonstrations, guided behavioral rehearsal, scenario-based learning, symptom-monitoring exercises, and practical self-care simulations. Content was designed to promote active participation and facilitate translation of educational material into daily self-management behaviors. For example, patients completing the behavioral activation module were guided through simulated daily planning activities that required organization of sleep routines, hygiene tasks, meal scheduling, physical activity, and social engagement. Emotion regulation modules included step-by-step demonstrations of breathing exercises, grounding techniques, and cognitive coping strategies that could be practiced during the session.

Each module required approximately 20–30 minutes to complete. Participants were encouraged to complete one module per session, with the full curriculum generally completed within two to four weeks depending on treatment setting and individual scheduling. A session was defined as a documented period of AR-based educational participation during which the patient completed or practiced at least one component of the curriculum under the guidance of the psychiatric nursing team. Modules could be revisited or repeated according to individual learning needs, and therefore the number of sessions was not necessarily equivalent to the number of unique curriculum modules completed.

Psychiatric nurses were responsible for introducing the intervention, orienting patients to the platform, monitoring participation, reviewing progress reports, and incorporating identified learning needs into individualized nursing care plans and discharge preparation. The educational content itself was standardized and delivered digitally to ensure consistency across participants.

Exposure to the AR-supported program was determined from documented participation in the hospital’s structured AR curriculum. The program was offered as an adjunct to routine psychiatric nursing education during the study period rather than being randomly assigned. Participation depended on clinical feasibility, patient willingness, and availability of the program within the relevant treatment setting. Thus, access to the program was not determined solely by clinical eligibility, and patients who declined participation or could not access the program remained eligible for the standard-care group. Patients who participated in and completed the structured AR curriculum were classified as the AR-supported group, whereas patients who received routine psychiatric nursing education without participation in the AR curriculum were classified as the standard-care group. Accordingly, exposure classification was based on documented completion rather than program initiation. Because exposure classification required completion of the curriculum, the possibility of completion-related selection bias was considered. Patients who completed the program may have differed from nonparticipants with respect to motivation, treatment engagement, clinical stability, or opportunity to participate. Exposure status was therefore established after completion of the curriculum rather than at the time of program initiation, and the potential for selection related to intervention completion was considered in the interpretation of the findings. The number of completed AR sessions was additionally recorded to characterize intervention exposure and to examine a potential dose-response relationship.

Routine psychiatric treatment, including pharmacotherapy, nursing care, psychoeducation, and psychosocial interventions, continued according to institutional protocols in both groups. The AR program was implemented as an adjunctive educational intervention and did not replace any component of standard treatment.

To evaluate potential dose-response effects, the number of completed AR sessions was additionally recorded and examined as a continuous exposure variable in secondary analyses.

### 2.3 Outcome measures

Outcome data were obtained from routine clinical assessments documented within the electronic medical record (EMR) system. For all participants, baseline assessments were collected at admission (inpatients) or initial outpatient evaluation, and follow-up assessments were obtained at discharge or the final documented clinical visit within the study period. Follow-up duration was calculated as the number of days between the baseline assessment and the endpoint assessment. Follow-up duration was summarized by treatment group and clinical setting to assess potential differences in observation time. Because follow-up duration was comparable between exposure groups, no separate adjustment for follow-up duration was applied in the primary paired analyses. Duration of care was retained as a covariate in the propensity score and multivariable regression models to account for variation in treatment exposure and observation time.

The primary clinical outcome was change in depressive symptom severity, assessed using the Chinese version of the 17-item Hamilton Depression Rating Scale (HAM-D-17) (17). The HAM-D-17 is one of the most widely used clinician-rated measures of depression severity in psychiatric practice. Total scores range from 0 to 52, with higher scores indicating more severe depressive symptoms. Change scores were calculated by subtracting follow-up scores from baseline scores, with larger positive values reflecting greater symptom improvement.

The primary functional outcome was change in self-care agency, assessed using the validated Mandarin Chinese version of the Exercise of Self-Care Agency (ESCA) Scale (18). The ESCA is a 43-item self-report instrument designed to evaluate an individual’s capacity to identify, initiate, and maintain self-care behaviors. Items are rated on a five-point Likert scale ranging from 1 (“very uncharacteristic of me”) to 5 (“very characteristic of me”), resulting in total scores ranging from 43 to 215. Higher scores indicate greater self-care agency. ESCA improvement was calculated as endpoint ESCA minus baseline ESCA, with positive values indicating greater improvement.

Secondary outcomes included medication adherence and selected healthcare utilization indicators. Medication adherence was assessed using routinely collected clinical documentation. For hospitalized patients, adherence was determined using Medication Administration Records (MARs) verified by ward nursing staff. For outpatients, adherence was evaluated using clinician-documented self-reports supplemented by pill-count information when available during follow-up visits. For the present analysis, adherence was classified according to the hospital’s routine quality-monitoring criterion, with patients showing less than 5% documented non-adherence during the observation period categorized as adherent. Thus, the adherent category could include patients with a small amount of documented non-adherence. Because adherence data were derived from different sources across care settings, clinical setting (inpatient versus outpatient) was included as a covariate in adjusted analyses. In addition, subgroup analyses were performed to examine whether observed associations were consistent across settings.

Healthcare utilization outcomes included length of stay (LOS) for the inpatient subgroup. LOS was calculated as the total number of hospitalization days from admission to discharge. Length of stay was analyzed as an exploratory outcome in the matched inpatient subgroup using a paired-samples t-test, with mean paired differences, 95% confidence intervals, and Cohen’s dz reported.

All outcome measures were obtained as part of routine clinical care and were recorded independently of the present study. Clinicians responsible for patient care were not aware of future research analyses at the time assessments were completed. For the purposes of this study, data extraction was performed using de-identified records and outcome analyses were conducted after removal of personal identifiers.

The use of routinely collected clinical data allowed evaluation of intervention performance under real-world practice conditions; however, variability in documentation practices and differences in adherence measurement across treatment settings were considered when interpreting study findings.

### 2.4 Bias control and blinding

Given the retrospective observational design, randomization and blinding of clinical personnel during treatment delivery were not feasible. Several measures were implemented to reduce the potential impact of selection, detection, and analytical bias.

To minimize detection bias, all study data were extracted from the electronic medical record system after completion of clinical care. Data extraction, database preparation, and statistical analyses were conducted by an investigator who was not involved in patient treatment. Prior to analysis, all records were de-identified and study group labels were masked until completion of the primary analyses.

Because participation in the AR-supported educational program was not randomly assigned, baseline differences between groups were anticipated. To reduce potential confounding, 1:1 nearest-neighbor propensity score matching without replacement was used to create a balanced analytic cohort. Propensity scores representing the probability of participation in the AR-supported program were estimated using a logistic regression model incorporating age, sex, education level, marital status, clinical setting (inpatient versus outpatient), duration of illness, duration of care, baseline HAM-D score, and baseline ESCA score. Psychotropic medication class and psychotherapy participation were not included in the propensity score model because the matching model was restricted to demographic, clinical, and baseline outcome characteristics; these treatment-related variables were instead examined descriptively in the matched cohort as potential sources of residual confounding.

Propensity score matching was performed using a one-to-one nearest-neighbor algorithm without replacement, with a caliper width of 0.20 standard deviations of the logit of the propensity score. Patients who could not be adequately matched within the specified caliper were excluded from the matched analyses. All primary outcome analyses were subsequently conducted using the propensity score–matched cohort.

Balance between exposure groups was evaluated using standardized mean differences (SMDs), with values below 0.10 considered indicative of acceptable covariate balance. Multivariable outcome models additionally included prespecified clinically relevant covariates to assess the robustness of the associations after matching. Although these procedures were implemented to reduce observed confounding, the possibility of residual confounding due to unmeasured variables, including motivation, digital literacy, illness insight, and treatment engagement, cannot be completely excluded and was considered during interpretation of findings.

### 2.5 Statistical analysis

Statistical analyses were conducted using IBM SPSS Statistics version 26.0. Descriptive statistics were used to summarize demographic and clinical characteristics. Continuous variables were reported as means and standard deviations, whereas categorical variables were summarized using frequencies and percentages. Patterns of missing data were evaluated using Little’s Missing Completely at Random (MCAR) test. Because overall missingness was low (<5%) and no systematic pattern of missing data was identified, complete-case analysis was performed.

Distributional assumptions were assessed using visual inspection of histograms and Q–Q plots in conjunction with the Shapiro–Wilk test. Multicollinearity among predictor variables was evaluated using Variance Inflation Factors (VIFs), with values greater than 5 considered indicative of potential concern. Baseline characteristics were compared between exposure groups before propensity score matching using independent-samples t-tests for continuous variables and chi-square tests for categorical variables. After propensity score matching, between-group differences in continuous outcomes were evaluated using paired t-tests to account for the matched-pair structure. Mean paired differences with 95% confidence intervals were reported. Binary outcomes were compared using McNemar’s test for matched pairs. For continuous outcomes analyzed using paired-samples t-tests, standardized effect sizes were expressed as Cohen’s dz, calculated as the mean paired difference divided by the standard deviation of the paired differences. Primary outcome analyses examined depressive symptom reduction (HAM-D) and improvement in self-care agency (ESCA).

Multivariable linear regression models were constructed within the propensity score-matched cohort. To account for the dependence introduced by matched pairs, cluster-robust standard errors were estimated using Pair_ID as the clustering variable.

Models included prespecified clinically relevant covariates, including age, sex, education level, duration of illness, duration of care, baseline outcome scores, and clinical setting. These analyses were performed to assess the robustness of the observed associations after propensity score matching. Medication adherence was analyzed as a binary outcome (full adherence versus partial/non-adherence). Because the primary analysis used the propensity score–matched cohort, differences in adherence between groups were evaluated using the exact McNemar test. The matched odds ratio was calculated from the discordant matched pairs; when a discordant cell was zero, the odds ratio was considered unbounded and an exact 95% confidence interval was reported.

Potential effect modification by treatment setting was explored through inclusion of an interaction term between intervention status and clinical setting. Because no statistically significant interaction was identified, pooled analyses were retained as the primary analytic approach. Within the AR-supported group, dose-response relationships were examined using Pearson correlation coefficients between the number of completed sessions and changes in outcome measures. Exploratory subgroup analyses were conducted separately for inpatient and outpatient participants. Length of stay was analyzed as an exploratory outcome among inpatients using a paired-samples t-test. All statistical tests were two-tailed, and statistical significance was defined as p < 0.05.

## 3 Results

During the study period, 417 patient records with a documented diagnosis of Major Depressive Disorder (MDD) were screened for eligibility. Seventy-five records were excluded because of incomplete outcome assessments (n = 28), comorbid psychotic disorders (n = 17), active substance dependence within the previous six months (n = 12), receipt of electroconvulsive therapy during the study period (n = 9), neurological conditions affecting cognitive functioning (n = 6), or substantial missing data in key study variables (n = 3). Overall missingness was below 5%, and Little’s MCAR test was not statistically significant, providing no evidence against the assumption of missing completely at random (MCAR).

The final analytic sample consisted of 342 patients. Of these, 199 (58.2%) received treatment in inpatient psychiatric units and 143 (41.8%) were managed through outpatient services. A total of 158 patients (46.2%) completed the AR-supported self-care program, whereas 184 patients (53.8%) received standard psychiatric nursing education without completion of the AR curriculum.

Baseline demographic and clinical characteristics are presented in **Table 1**. Before propensity score matching, participants in the AR-supported group were younger than those in the standard-care group (35.78 ± 8.23 vs. 38.23 ± 7.58 years; t = −2.86, p = .005). University-level education was also more common among AR participants (48.73% vs. 38.04%; χ^2^ = 3.96, p = .046). No statistically significant between-group differences were observed for sex, marital status, treatment setting, duration of illness, duration of care, baseline HAM-D score, or baseline ESCA score.

**Table 1:** Baseline demographic and clinical characteristics before propensity score matching (N = 342).

Characteristic	AR-Supported (n = 158)	Standard Care (n = 184)	Test	p-value
Age (years), Mean ± SD	35.78 ± 8.23	38.23 ± 7.58	t = −2.86	.005
Female, n (%)	95 (60.1)	104 (56.5)	χ^2^ = 0.45	.500
Married, n (%)	97 (61.4)	117 (63.6)	χ^2^ = 0.17	.676
University education, n (%)	77 (48.73%)	70 (38.04)	χ^2^ = 3.96	.046
Inpatient, n (%)	94 (59.5)	105 (57.1)	χ^2^ = 0.21	.650
Duration of care (days), Mean ± SD	18.8 ± 6.1	19.1 ± 7.0	t = −0.43	.668
Duration of illness (months), Mean ± SD	16.3 ± 7.5	15.91 ± 8.17	t = 0.45	.653
Baseline HAM-D, Mean ± SD	23.39 ± 3.1	23.33 ± 3.0	t = 0.18	.856
Baseline ESCA, Mean ± SD	48.43 ± 9.06	49.27 ± 8.45	t = −0.88	.378

HAM-D, Hamilton Depression Rating Scale; ESCA, Exercise of Self-Care Agency Scale.

The observed baseline differences in age and educational attainment indicated potential imbalance between the exposure groups before adjustment. Therefore, age, education level, and other prespecified demographic and clinical characteristics were incorporated into the propensity score model to reduce observed baseline imbalance before outcome analyses.

Among the propensity score–matched cohort, the distribution of psychotropic medication classes did not differ significantly between groups (SSRI: 72 [49.7%] vs. 70 [48.3%]; SNRI: 53 [36.6%] vs. 46 [31.7%]; other psychotropic medications: 20 [13.8%] vs. 29 [20.0%]; χ^2^ = 2.18, p = .337). Psychotherapy participation was identical between groups (64/145 [44.1%] in each group; χ^2^ = 0.00, p = 1.000). Follow-up duration was comparable between groups in the matched cohort. The mean follow-up duration was 23.72 ± 6.56 days in the AR-supported group and 23.85 ± 6.80 days in the standard-care group. Among inpatients, mean follow-up duration was 20.36 ± 4.33 versus 20.86 ± 5.12 days, respectively, whereas among outpatients it was 28.34 ± 6.29 versus 28.46 ± 6.52 days, respectively. After propensity score matching, all prespecified matching covariates achieved acceptable balance, with absolute SMDs below 0.10. Standardized mean differences for all variables included in the propensity score model were below the prespecified threshold of 0.10, indicating adequate balance between the exposure groups (**Table 2**).

**Table 2:** Covariate balance before and after propensity score matching.

Variable	SMD before matching	SMD after matching
Age	0.309	0.025
Education level	0.217	0.000
Sex	0.073	0.056
Marital status	0.045	0.057
Clinical setting	0.049	0.056
Duration of illness	0.050	0.024
Duration of care	0.047	0.004
Baseline HAM-D	0.020	0.011
Baseline ESCA	0.095	0.007

Absolute standardized mean differences (SMDs) were calculated using pooled standard deviations for continuous variables and standardized differences in proportions for binary variables. An absolute SMD <0.10 was considered indicative of adequate covariate balance.

The propensity score distributions showed adequate overlap between the two exposure groups, and no clinically important violation of the common-support assumption was identified. A Love plot was used to display standardized mean differences before and after matching (**Figure 1**).

**Figure 1:**
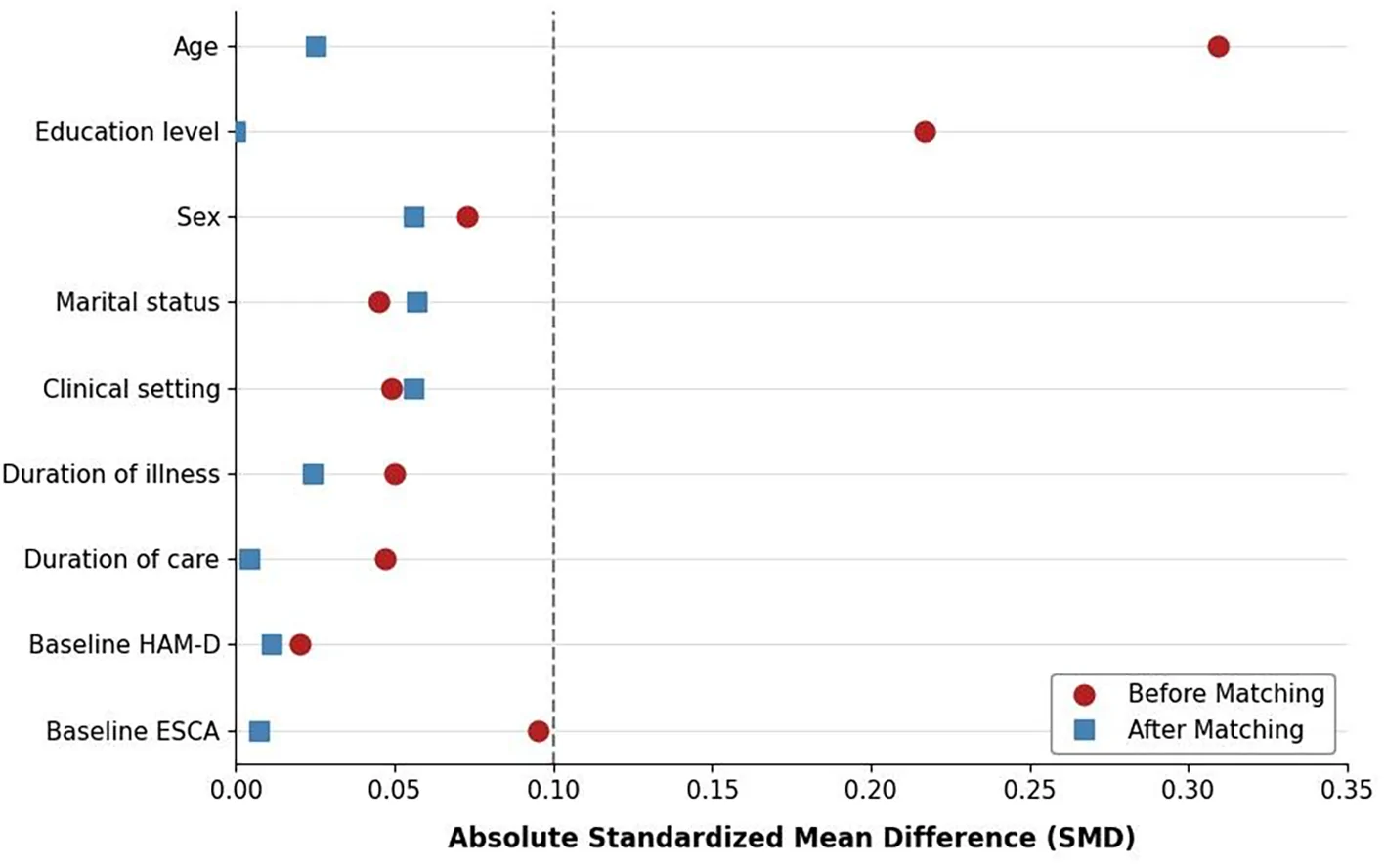
Covariate balance before and after propensity score matching; The Love plot displays the absolute standardized mean differences (SMDs) for all measured covariates before (red circles) and after (blue squares) propensity score matching. The vertical dashed line represents the prespecified threshold of 0.10. Following the matching procedure, the SMDs for all covariates were substantially reduced and fell well below the 0.10 threshold, indicating excellent balance between the AR-supported and standard-care groups.

After propensity score matching, 145 patients in the AR-supported group were matched with 145 patients in the standard care group. Of the 158 patients receiving the AR-supported program, 145 were successfully matched, whereas 13 remained unmatched because no eligible counterpart was identified within the prespecified caliper. Among 184 patients receiving standard care, 145 were matched and 39 were excluded from matched analyses because no adequate match was available. All matched pairs had complete Pair_ID linkage, and covariate balance was achieved with standardized mean differences below 0.10. Clinical and functional outcomes in the matched cohort are presented in **Table 3**.

**Table 3:** Clinical and functional outcomes in the propensity score-matched cohort.

Outcome	AR-supported	Standard care	Mean paired difference	95% CI	Effect size	p-value
Endpoint HAM-D	10.79 ± 3.84	12.31 ± 4.09	−1.52	−2.38 to −0.65	dz = −0.29	0.001
HAM-D reduction	12.54 ± 3.06	10.99 ± 3.28	1.55	0.81 to 2.29	dz = 0.34	<0.001
Endpoint ESCA	89.6 ± 12.84	80.67 ± 11.94	8.93	6.26 to 11.61	dz = 0.55	<0.001
ESCA improvement	40.86 ± 11.08	31.99 ± 9.02	8.87	6.61 to 11.12	dz = 0.64	<0.001
Full Adherence (%)	131/145 (90.3%)	119/145 (82.1%)	—	2.78 to ∞	∞	<0.001
Length of Stay (days)*^†^*	19.69 ± 4.59	21.40 ± 4.87	−1.71	−3.54 to 0.12	dz = −0.25	0.067

† Inpatient matched subgroup only (55 matched pairs). Continuous outcomes were analyzed using paired-samples t-tests, and standardized effect sizes are reported as Cohen’s dz, calculated from the mean paired difference and the standard deviation of the paired differences. Medication adherence was analyzed using the exact McNemar test. The matched odds ratio was based on discordant pairs. Because no discordant pair favored standard care (0 pairs), the matched odds ratio was unbounded; the confidence interval was calculated using an exact conditional method. Mean paired differences represent AR-supported minus standard-care values.

In the matched cohort, paired analyses demonstrated significantly greater improvements among patients receiving AR-supported education. Endpoint HAM-D-17 scores were lower in the AR-supported group compared with the standard-care group (10.79 ± 3.84 vs. 12.31 ± 4.09), corresponding to a mean paired difference of −1.52 points (95% CI: −2.38 to −0.66, p = 0.001). Reduction in HAM-D-17 scores was also greater in the AR-supported group (12.54 ± 3.06 vs. 10.99 ± 3.28), with a mean paired difference of 1.55 points (95% CI: 0.81 to 2.29, p < 0.001).

For functional outcomes, endpoint ESCA scores were significantly higher among AR-supported patients (89.60 ± 12.84 vs. 80.67 ± 11.94; mean paired difference = 8.93, 95% CI: 6.26 to 11.61, p < 0.001). Similarly, improvement in ESCA scores was greater in the AR-supported group (40.86 ± 11.08 vs. 31.99 ± 9.02; mean paired difference = 8.87, 95% CI: 6.61 to 11.12, p < 0.001).

Medication adherence was high in both groups. Full adherence was documented in 131 patients (90.3%) in the AR-supported group and 119 patients (82.1%) in the standard-care group. Among the 145 matched pairs, 119 pairs were concordantly adherent, 14 pairs were concordantly non-adherent, 12 pairs were discordant in favor of the AR-supported group, and no pairs were discordant in favor of standard care. Accordingly, the matched odds ratio was unbounded because the latter discordant cell was zero; the exact 95% confidence interval had a lower bound of 2.78. Because adherence was assessed within matched pairs, the difference between groups was evaluated using the exact McNemar’s test, which demonstrated a statistically significant difference favoring the AR-supported group (p < 0.001). In exploratory subgroup analyses, full adherence was documented in 74 of 84 AR-supported inpatients (88.1%) compared with 68 of 88 matched standard-care inpatients (77.3%), and in 57 of 61 AR-supported outpatients (93.4%) compared with 51 of 57 matched standard-care outpatients (89.5%). These subgroup findings were interpreted cautiously because of the smaller sample size. Among the matched inpatient subgroup (55 pairs), the mean length of stay was 19.69 ± 4.59 days in the AR-supported group and 21.40 ± 4.87 days in the standard-care group. The mean paired difference was −1.71 days (95% CI, −3.54 to 0.12; p = .067). This analysis was considered exploratory because length of stay was restricted to hospitalized patients and may be influenced by administrative, discharge-planning, and healthcare-system factors.

Within the AR-supported group, patients completed a median of five sessions (interquartile range: 4–6 sessions). A significant positive association was observed between the number of completed AR sessions and improvement in self-care agency scores (r = 0.29, p < 0.001). Patients with greater exposure to the intervention tended to demonstrate larger gains in self-care capacity. The association between the number of completed AR sessions and HAM-D reduction was weak and not statistically significant (r = -0.10, p = 0.223). Exploratory subgroup analyses demonstrated similar patterns in both inpatient and outpatient settings. No statistically significant interaction between AR participation and treatment setting was observed for ESCA improvement (interaction p = 0.814). A similar lack of interaction was observed for HAM-D reduction (interaction p = 0.657).

To evaluate the robustness of the primary findings after propensity score matching, multivariable regression analyses were performed within the matched cohort (**Table 4**). For depressive symptom improvement, the regression model was statistically significant (F = 3.85, p < 0.001), explaining 7.3% of the variance in HAM-D score reduction (adjusted R^2^ = 0.073). Participation in the AR-supported program remained significantly associated with greater symptom improvement after adjustment for the prespecified covariate differences (β = 0.24, p < 0.001). Baseline HAM-D score was also a significant predictor of symptom reduction (β = 0.19, p = 0.001). No significant associations were observed for age, sex, education level, treatment setting, duration of illness, or duration of care.

**Table 4:** Multivariable regression models in the propensity-matched cohort.

Predictor	HAM-D reduction β	95% CI	p-value	ESCA improvement β	95% CI	p-value
AR participation	0.24	[0.13, 0.35]	<0.001	0.40	[0.30, 0.50]	<0.001
Age	-0.05	[-0.17, 0.07]	0.406	0.08	[-0.04, 0.20]	0.175
Female sex	0.00	[-0.11, 0.11]	0.942	-0.06	[-0.16, 0.04]	0.254
University education	0.02	[-0.14, 0.18]	0.806	0.09	[-0.02, 0.20]	0.101
Baseline HAM-D/ESCA	0.19	[0.08, 0.30]	0.001	-0.11	[-0.22, -0.01]	0.048
Inpatient setting	-0.04	[-0.15, 0.07]	0.467	-0.06	[-0.16, 0.04]	0.261
Duration of illness	0.04	[-0.07, 0.15]	0.461	0.00	[-0.10, 0.10]	0.975
Duration of care	-0.01	[-0.15, 0.13]	0.890	0.01	[-0.17, 0.19]	0.914

β represents standardized regression coefficients. Baseline HAM-D and baseline ESCA scores were entered into the respective outcome models.

For self-care outcomes, the model accounted for 17.6% of the variance in ESCA score improvement (F = 8.73, p < 0.001; adjusted R^2^ = 0.176). AR participation remained significantly associated with greater improvement in self-care agency (β = 0.40, p < 0.001). Baseline ESCA score demonstrated a significant inverse association with subsequent improvement (β = -0.11, p = 0.048), indicating that patients with lower initial self-care capacity experienced greater gains. Age, sex, education level, treatment setting, duration of illness, and duration of care were not significant predictors. Variance Inflation Factors ranged from 1.00 to 1.15, indicating no evidence of problematic multicollinearity.

## 4 Discussion

This retrospective propensity score–matched cohort study evaluated the association between participation in an augmented reality (AR)-supported self-care training program and clinical outcomes among patients receiving routine psychiatric nursing care for Major Depressive Disorder (MDD). After balancing measured baseline characteristics between groups, participation in the AR program remained associated with greater improvement in self-care agency, modestly greater reduction in depressive symptoms, and higher rates of medication adherence. These findings suggest that AR-supported educational approaches may provide added value when integrated into existing psychiatric nursing services, particularly in domains related to self-management and functional recovery.

The strongest association observed in the present study involved self-care agency. Although both groups demonstrated improvement over the course of treatment, patients in the AR-supported group demonstrated greater improvement in self-care agency. This finding is consistent with experiential learning frameworks, which propose that active participation and contextualized practice facilitate acquisition of behavioral skills more effectively than passive information delivery alone (19). Rather than relying exclusively on verbal explanations or printed educational materials, the AR platform allowed patients to interact with visual prompts and simulated real-world situations relevant to symptom management and daily functioning. Such approaches may be particularly relevant for individuals with MDD, whose attentional and executive functioning difficulties can limit engagement with traditional educational strategies (12, 13).

The observed relationship between intervention exposure and self-care improvement further supports this interpretation. Although the correlation was modest, patients who completed a greater number of AR sessions generally demonstrated larger improvements in ESCA scores. Importantly, this association was evident primarily for functional outcomes rather than symptom reduction. This pattern suggests that AR-supported education may be particularly relevant to practical self-management skills, whereas changes in depressive symptom severity may be influenced by a broader range of treatment and illness-related factors beyond the educational intervention itself. For self-care outcomes, lower baseline ESCA scores were associated with greater subsequent improvement, while age, sex, education level, treatment setting, duration of illness, and duration of care were not significant predictors. From a nursing perspective, this distinction is important because functional recovery and symptom remission do not always occur simultaneously (20, 21).

Because adherence was measured through routine clinical documentation rather than electronic monitoring systems, these findings should be interpreted cautiously. Although adherence was generally high across both groups, the observed difference may be clinically relevant given the established relationship between adherence, relapse prevention, and long-term treatment outcomes in MDD. One possible explanation is that the interactive educational content may have supported patients’ understanding of treatment goals and the importance of medication adherence (22). Previous digital health research has suggested that visual and interactive educational formats can enhance engagement and treatment understanding, particularly among individuals with chronic health conditions (22, 23). However, because medication management practices were not experimentally controlled in the present study, this interpretation should be considered exploratory.

The association between AR participation and depressive symptom improvement was statistically significant but comparatively modest. This finding may be clinically meaningful because the intervention was not designed as a psychotherapeutic treatment targeting depressive symptoms directly. Rather, the AR curriculum focused primarily on education, self-care behaviors, emotion regulation strategies, and daily functioning. Consequently, the modest additional improvement observed in HAM-D scores may reflect indirect pathways related to improved self-management and treatment adherence, although these pathways were not directly tested in the present study. The multivariable analysis also indicated that greater baseline depressive symptom severity was associated with greater subsequent symptom reduction, whereas age, sex, education level, treatment setting, duration of illness, and duration of care were not significant predictors. Future prospective studies incorporating mediation analyses could help clarify these relationships.

An important finding was the absence of evidence that the association between AR participation and outcomes differed by treatment setting. No significant interaction was observed between treatment setting and intervention exposure, suggesting that the observed association between AR participation and outcomes did not differ significantly between inpatient and outpatient settings. This observation is consistent with growing interest in technology-assisted approaches that support continuity of care across treatment transitions (24). For psychiatric nurses, digital educational platforms may offer opportunities to reinforce self-management skills beyond individual encounters and to provide more standardized educational experiences across clinical environments (25).

At the same time, the study highlights important equity considerations. Prior to propensity score matching, participants in the AR-supported group were younger and more likely to have a university-level education. These differences were substantially reduced after matching, and neither age nor education level was significantly associated with outcome improvement in the subsequent multivariable analyses. Nevertheless, the pre-matching differences suggest that access to and uptake of technology-based educational interventions may vary across patient characteristics. Although digital literacy was not directly assessed in the present study, future implementation efforts should consider accessibility and provide appropriate support for patients who may have greater difficulty using digital technologies (26). Future implementation efforts should therefore consider user-centered design principles, simplified interfaces, and structured nursing support to promote equitable access.

Several limitations should be considered when interpreting these findings. First, the retrospective observational design precludes causal inference. Although propensity score matching improved comparability between groups and reduced measured confounding, residual confounding from unmeasured factors remains possible. Variables such as intrinsic motivation, readiness for behavioral change, family support, socioeconomic status, and digital literacy were not routinely captured within the electronic medical record and therefore could not be incorporated into matching procedures. Second, the study was conducted at a single tertiary psychiatric hospital, which may limit generalizability to other healthcare systems and cultural contexts. Psychiatric nursing roles, patient education practices, and technology adoption patterns vary considerably across countries and healthcare settings. In addition, eligibility was restricted to adults aged 18–60 years, which limits generalizability to older adults who may differ in digital literacy, accessibility needs, cognitive functioning, and engagement with AR-supported interventions. Third, medication adherence was assessed using different documentation procedures in inpatient and outpatient settings, which may have introduced measurement variability despite efforts to standardize outcome definitions. In addition, AR exposure was defined on the basis of documented completion of the structured curriculum, which may have introduced completion-related selection bias. Patients who completed the program may have differed from nonparticipants in motivation, treatment engagement, clinical stability, or opportunity to participate, factors that could not be fully addressed through propensity score matching. Although the distributions of psychotropic medication classes and psychotherapy participation were similar between groups in the matched cohort, residual confounding related to differences in treatment intensity or other unmeasured aspects of concomitant psychiatric care cannot be excluded. Because exposure was defined according to completion of the structured AR curriculum, patients who initiated the program but discontinued before completing the curriculum could not be separately evaluated in the present analytic dataset. Therefore, an initiation-based sensitivity analysis could not be performed. This completion-based exposure definition may have preferentially selected patients with greater treatment engagement, motivation, clinical stability, or opportunity to participate. Accordingly, the observed associations should not be interpreted as estimates of the effect that would have been observed among all patients initially offered or initiating the AR program. Finally, follow-up was limited to the active treatment period, preventing evaluation of whether observed improvements in self-care behaviors were sustained after discharge or completion of outpatient care. The duration of care also varied across participants, although it was recorded and included as a covariate in adjusted analyses.

Despite these limitations, the study contributes to the emerging literature on digital innovations in psychiatric nursing by examining AR implementation under routine clinical conditions rather than highly controlled research environments. The findings suggest that AR-supported self-care education may represent a potentially valuable adjunct to standard psychiatric nursing practice, particularly for supporting self-management and functional recovery among individuals with MDD.

## 5 Conclusion

In this retrospective propensity score–matched cohort study, participation in an AR-supported self-care training program was associated with greater improvement in self-care agency, higher medication adherence, and modestly greater reduction in depressive symptoms among patients receiving routine psychiatric nursing care for Major Depressive Disorder. The strongest associations were observed for functional outcomes related to self-management rather than symptom reduction alone.

These findings suggest that AR-based educational tools may complement traditional psychiatric nursing interventions by providing interactive and contextually relevant opportunities for patients to practice self-care skills. However, given the observational nature of the study, the results should be interpreted as evidence of association rather than causation. Further prospective, multicenter studies are needed to determine whether these findings can be replicated across diverse populations and healthcare settings and to evaluate the long-term sustainability of observed benefits.

For psychiatric nursing practice, AR-supported education may represent a promising strategy for enhancing patient engagement and supporting functional recovery. Future implementation efforts should consider digital literacy, accessibility, and equitable participation to ensure that potential benefits are available across diverse patient populations.

Randomized controlled trials are warranted to determine whether the observed associations represent true causal effects and to identify the patient populations most likely to benefit from AR-supported psychiatric nursing interventions.

## Data Availability

The raw data supporting the conclusions of this article will be made available by the authors, without undue reservation.
